# Effects of dietary glycerol inclusion on growth performance, carcass and meat quality characteristics, glycogen content, and meat volatile compounds in Korean cattle steers

**DOI:** 10.5713/ajas.20.0186

**Published:** 2020-06-24

**Authors:** Minyu Piao, Da Jin Sol Jung, Hyeok Joong Kang, Seung Ju Park, Jin Oh Lee, Minsu Kim, Hyun Jin Kim, Do Hyun Kim, Ja Kyeom Seo, Cheorun Jo, Md Najmul Haque, Myunggi Baik

**Affiliations:** 1 Department of Agricultural Biotechnology and Research Institute of Agriculture and Life Sciences, College of Agriculture and Life Sciences, Seoul National University, Seoul 08826, Korea; 2 Feed Research Institute, Chinese Academy of Agricultural Sciences, Beijing 100081, China; 3 Life and Industry Convergence Research Institute and Department of Animal Science, College of Natural Resources and Life Sciences, Pusan National University, Miryang 50463, Korea; 4 Institute of Green Bio Science and Technology, Seoul National University, Pyeongchang 25354, Korea

**Keywords:** Korean Cattle Steer, Glycerol, Feed Intake, Carcass and Sensory Traits, Glycogen Contents, Volatile Compounds

## Abstract

**Objective:**

We have tested our hypothesis that inclusion of purified glycerol as a replacer of portions of dried distillers grain with solubles (DDGS) would affect growth performance, rumen fermentation and blood parameters, carcass and sensory traits, reducing sugar and glycogen contents, and volatile compound profiles in *longissimus thoracis* (LT) in Korean cattle steers.

**Methods:**

A total of 20 Korean cattle steers (27.0±0.2 months old; 647±10.5 kg body weight [BW]) were assigned to a conventional control group or a glycerol group (3.17% purified glycerol addition as a replacement for DDGS and molasses). The steers were individually allowed to receive the experimental concentrate at the daily amount of 1.5% of their individual BW and a total 1.0 of kg/d of rice straw twice daily. The feeding trial was conducted for a period of 20 weeks.

**Results:**

Glycerol supplementation (GS) increased (p = 0.001) concentrate intake. However, GS did not affect (p>0.05) average daily gain, feed efficiency, and ruminal volatile fatty acid concentrations. GS tended to increase (p≤0.10) serum glucose concentrations at the 16th and 20th weeks. GS decreased (p = 0.001) LT pH. GS did not affect (p>0.05) carcass traits and the chemical or physicochemical compositions, reducing sugar or glycogen contents, sensory traits, and most of volatile compounds in the LT.

**Conclusion:**

The inclusion of purified glycerol as a replacement for DDGS in the finishing diet did not affect growth performance, rumen fermentation parameters, and carcass quality in Korean cattle. The purified glycerol could be used as a substitute for other energy sources such as DDGS in beef cattle, depending on the price.

## INTRODUCTION

Glycerol is generated during biodiesel production via catalyzed reactions between alcohol and triacylglycerides in animal fats and vegetable oils [1]. Previous studies have shown inconsistent results of effects of dietary glycerol inclusion on beef cattle; one of the studies showed no effect of crude glycerin supplementation on animal performance or carcass characteristics [2]. However, other studies have found that crude glycerin feeding improved weight gain and feed efficiency in grazing Nellore bulls [3], improved the overall acceptability of meat [4]. Most studies, including those cited above, were performed using crude glycerin containing methanol. Very few have been carried out using purified glycerol without methanol. For these reason, we tested the effects of purified glycerol inclusion on growth performance and carcass quality in cattle.

The production of distiller’s grains plus solubles (DGS), including dried distiller’s grains with solubles (DDGS), has increased as a result of the expansion of bioenergy ethanol production. This has made the use of DGS competitive with traditional energy sources as corn [5]. Glycerin may have an energy content similar to that of DGS or corn in finishing diets [5]. Previously, diets containing 15% glycerin in combination with 30% DDGS improved the growth performance of early-weaned beef calves and meat quality at slaughter [6]. Purified glycerol contains uniform energy levels, whereas DDGS has variations in nutrient compositions. Thus, purified glycerin inclusion may have an advantage for providing constant energy levels compared with DDGS. However, little information is available on the effects of the purified glycerol inclusion as a replacement for DDGS on performance and meat quality in beef cattle.

Glycerol potentially serves as a gluconeogenic substrate for ruminants [7]. In the rumen, glycerol can be fermented to propionate, which can act as a glucogenic precursor [8]. Glucose can be used as a carbon source for fatty acid synthesis [9,10], possibly contributing to an increase in the degree of beef marbling [10]. Glucose can also be stored as glycogen in the muscle and affect beef quality [11]. To our knowledge, whether dietary glycerol affects the beef glycogen content is unknown.

Flavor is an important factor in meat quality. Large quantities of volatile compounds, such as aldehydes, hydrocarbons, and ketones, are formed during the process of cooking meat, and these compounds influence meat flavor [12]. Reducing sugars such as glucose and ribose have been reported to improve the flavor of cooked meat by reacting with amino acids to produce many important volatile compounds via the Maillard reaction [12]. However, limited information is available regarding the effect of dietary glycerol on glucose production and volatile compound profiles in beef.

In this study, we tested our hypothesis that inclusion of the purified glycerol as a replacer of portions of DDGS would affect growth performance, rumen fermentation and blood parameters, carcass and sensory traits, reducing sugar and glycogen contents, and volatile compound profiles in *longissimus thoracis* (LT) in Korean cattle steers.

## MATERIALS AND METHODS

### Animal care

The experimental procedures were approved by the Seoul National University (SNU) Institutional Animal Care and Use Committee (SNU-151208-2).

### Animals, diets, and chemical analyses

Twenty Korean cattle steers were reared at the University Animal Farm of the College of Agriculture and Life Sciences, Pyeongchang Campus of SNU in South Korea. In order to assign the cattle uniformly into two groups, estimated marbling scores were obtained by ultrasound. Based on body weight (BW), age, and the estimated marbling score, the steers were assigned to one of two dietary treatments: a control concentrate group and a glycerol concentrate group (replacing the DDGS portion with 3.17% purified glycerol). The control concentrate contained 0.4% glycerol. The glycerol-containing concentrate was prepared by adding purified glycerol adsorbed to ground wheat bran (63% glycerol adsorbed to 37% ground wheat bran) to achieve a final 3.57% of glycerol during the concentrate pelleting process. The purified glycerol (99.7% glycerol and 0.3% water) was obtained from Palm Oleo Sdn. Bhd. (Selangor, Malaysia). In our previous study, the 2% glycerol addition did not affect serum glucose concentrations of Korean cattle steers [13]. Glycerin inclusion at 2% and 4% improved desirable fatty acid percentages in intramuscular fat and juiciness of beef in Limousin bull [14]. In this study, we thus selected 3.17% higher glycerol levels compared to control group with the consideration of composition of other ingredients in the pelleted diet. In the current study, we used a fixed level of glycerol inclusion because we had only limited number of animals, which were not sufficient to test several levels of replacement. The DDGS was partially replaced with glycerol to ensure that the two diets were isocaloric. The molasses (3.62%) contained in the control concentrate provides viscosity and aids pellet properties, and the molasses levels in the glycerol diet was thus reduced to 0.91% since glycerol addition increases viscosity and can change the pellet properties. Replacement of glycerol for molasses also aids in making the control and glycerol diets isocaloric and balanced the carbohydrate intakes between the two diets. The ingredients and chemical compositions of the diets are presented in Table 1. The pelletized forms of the control concentrate and the glycerol-containing concentrate were prepared by Cargill Agri Purina, Inc. (Seongnam, Korea).

During a 2-week adaptation period, all animals were fed the control concentrate and rice straw. The animals were then weighed, and the experiment was started with an initial BW of 647±10.5 kg and at 27.0±0.2 months of age. In Korean cattle steers, 27 months of age is in the late fattening period, which is the relatively slow growing phase. However, intramuscular fat has been continuously deposited in this stage [15]. The steers were individually allowed to receive the experimental concentrate at the daily amount of 1.5% of their individual BW using an automatic feeding station (Dawoon system, Incheon, Korea), and the daily concentrate intake was recorded automatically using a computer system. The animals were individually fed a total 1.0 of kg/d of rice straw twice daily at 08:00 am and 17:00 pm, and the residual roughage was weighed before the morning feeding. The animals had free access to water and mineral blocks. The experimental feeding periods were 20 weeks in duration. BW was measured at 09:00 am before feeding at 4-week intervals. Samples of concentrates and rice straw were collected at 2-week intervals and stored at −20°C until analysis. Fecal samples were collected by fecal grab at 4-week intervals, and both diet and fecal samples were ground and stored at −20°C until analysis.

The chemical composition (dry matter [DM], crude protein, ether extract, ash, and Ca) of feed and fecal samples was determined according to AOAC methods [16], and neutral detergent fiber, acid detergent fiber, and lignin were determined according to the method of Van Soest et al [17]. Total digestible nutrients and metabolizable energy were calculated based on NRC [18]. Nutrient digestibility was estimated using the indirect digestibility method with the lignin ratio technique described by Wallace and Van Dyne [19]. Briefly, the lignin, a naturally-occurring plant constituent, was assumed as an indigestible chemical component, and it was used as an indicator in the process of calculating the digestibility of a nutrient in feed with following equations:

$$\text{Digestibility of a nutrient }(\%)=100-100\times \left(\frac{\%\;\text{indicator in feed consumed}}{\%\;\text{indicator in feces}}\times \frac{\%\;\text{nutrient in feces}}{\%\;\text{nutrient in feed consumed}}\right)$$

### Blood and rumen fluid collection and measurements

Blood was collected at 3 h post-feeding at 4-week intervals by jugular venipuncture and transferred into non-heparinized vacutainers (20 mL; Becton-Dickinson, Franklin Lakes, NJ, USA) for serum and ethylenediaminetetraacetic acid-treated vacutainers for plasma (20 mL). Both serum and plasma were separated by centrifugation at 1,500×*g* at 4°C for 15 min and stored at −80°C until analysis. The serum was used for glucose analysis. Reagents for the glucose analysis were purchased from JW Medical (Seoul, Korea). Glucose was analyzed using an automated chemistry analyzer (Hitachi 7180; Hitachi, Tokyo, Japan). Plasma ghrelin was analyzed using a bovine ghrelin ELISA kit (MBS013058; MyBioSource, San Diego, CA, USA). The intra-and inter-assay coefficients of variation for the ghrelin kit were both less than 15%.

Rumen fluid samples were harvested at 3.5 h post-feeding using the oral-stomach tube method as described by Shen et al [20]. The rumen fluid pH was measured with a pH meter (Ohaus Corp., Parsippany, NJ, USA). For volatile fatty acid (VFA) analysis, 1 mL of rumen fluid was mixed with 0.2 mL of 25% meta-phosphoric acid and the mixture stored at −20°C until analysis. A portion of the rumen fluid for NH_3_-N analysis was also stored at −20°C until analysis. The VFA and NH_3_-N concentrations were determined as described previously [21]. Briefly, the NH_3_-N concentration was determined using a modified colorimetric method [22], and the VFA concentrations were determined by gas chromatography using an Agilent Tech 7890A (Agilent Technologies, Waldbronn, Germany) for which a Supelco fused silica capillary column (30 m×0.25 mm×0.25 μm) was used.

### Slaughter procedures, carcass measurements, and tissue sample collections

Animals were transported for 4 hours to a local municipal slaughterhouse (Bucheon, Korea), and slaughtered the following day. After undergoing captive-bolt stunning, the animals were slaughtered in a conventional manner as reported previously [23]. Immediately after slaughter, LT samples were taken from between the 12th and 13th rib of the warm carcass, frozen in liquid nitrogen, and stored at −80°C until glycogen analysis.

At 24 h post-mortem, the carcasses were evaluated by the Korean carcass-grading system of the Korea Institute for Animal Products Quality Evaluation [24]. Carcass characteristics for meat quality and yield grades were determined by an official meat grader as described by Piao et al [25]. The LT (cold carcass weight: about 1 kg) was obtained from the 12th vertebra, vacuum-packaged, and transported on ice to a laboratory. After transportation to the laboratory, the packages containing the LT samples were opened and the external fat was trimmed away. The LT samples were minced using a mini chopper (CH180, Kenwood, Shanghai, China) for 30 s. Minced LT samples from various locations were pooled, and some samples were used immediately for the evaluation of pH and chemical composition, whereas others were stored at −70°C for the analysis of reducing sugar and volatile compound profiles. Samples for shear force, meat color (Commission Internationale de l’Eclairage [CIE] value), and sensory trait analyses were collected but not minced, and shear force and meat color were immediately determined, whereas samples destined for sensory evaluation were stored at −70°C for 1 week.

### Chemical and physiochemical compositions of the *longissimus thoracis*

Moisture, crude protein and crude fat content were determined according to AOAC methods [16]. Surface-color values (CIE; L*, a*, and b* values represent lightness, redness, and yellowness, respectively) were measured using a colorimeter (CM-5, Minolta Co., Ltd., Osaka, Japan) as described by Piao et al [26]. The pH of the beef samples was measured using a pH meter (SevenGo, Mettler-Toledo Inti., Inc., Schwerzenbach, Switzerland) as described by Piao et al [27]. The shear force value (kg) was measured using a Warner-Bratzler shear attached to a texture analyzer (CT3 10K, Brookfield Engineering Laboratories., Middleboro, MA, USA) as described by Piao et al [25].

### Reducing sugar and glycogen content of the *longissimus thoracis*

The LT reducing sugar content was measured using the dinitrosalicylic acid method as described by Piao et al [26].

To analyze the glycogen, ground meat (2 g) was suspended in 8.5% perchloric acid (10 mL) and homogenized at Lv. 6 for 30 s (T10, Ika Works, Staufen, Germany). The homogenate was centrifuged (Combi 514R, Hanil Co., Ltd., Incheon, Korea) and filtered through glass wool. The pellet was re-extracted with 8.5% perchloric acid, and supernatants were obtained in the same manner. Iodine color reagent (a solution containing iodine and potassium iodide with calcium chloride) was added to the glycogen extracts, and the absorbance at 460 nm was measured (X-ma 3100, Human Co., Ltd., Seoul, Korea). The amount of glycogen was calculated using a standard curve developed for glycogen (Sigma-Aldrich, St. Louis, MO, USA).

### Sensory analyses of the *longissimus thoracis*

Human ethics approval for sensory analyses were granted by Seoul National University Institutional Review Board (SNU 19-04-040). A total of 20 LT samples (n = 10 for each treatment) were evaluated during nine different sessions. Eleven panelists (five men and six women ranging in age from 28 to 34 years) were participated in the sensory evaluation. All sessions were conducted in the Animal Origin Food Science Laboratory at Seoul National University. For the sensory evaluation, the samples were randomly coded with three-digit-number and cut into the same size (15×40×15 mm^3^). Then, the LT samples were cooked until their internal temperature were reached to 72°C. During cooking, their internal temperature was monitored using a digital thermometer (YF-160A Type-K; YFE, Hsinchu, Taiwan) that was placed in the center of the LT samples. After the completion of cooking, the cooked meat was maintained at 72°C before serving and served to the panel with sufficient water to cleanse their palate between the samples. The panel evaluated the appearance, odor, taste, flavor, tenderness, juiciness, and overall acceptability of the cooked samples using a 9-point hedonic scale, (1 = dislike extremely to 9 = like extremely).

### Volatile compound profiles of the *longissimus thoracis*

Volatile compounds were analyzed as described by Piao et al [26]. Briefly, LT samples were grilled on a hot plate (PC-420D; Corning, NY, USA) until they reached an internal temperature of 72°C and transferred to glass vials (N9306078; PerkinElmer, Boston, MA, USA). The vials were placed in the oven of a headspace sampler, and extraction of the volatile compounds of the samples was performed using a headspace auto-sampler. The transfer line from the headspace sampler was directly connected to the injector for the gas chromatograph (GC). A Perkin Elmer 680 gas chromatograph equipped with a 600T mass spectrometry detector was used to analyze the volatile compounds. The compounds were separated using an HP-PLOT Q column (Agilent, Wilmington, DE, USA; 30 m×0.53 mm×0.25-μm film thickness). The resolved mass spectrometry spectra obtained from custom scripts were matched against reference mass spectra using the National Institute of Standards and Technology (NIST) mass spectral search program and the NIST/US Environmental Protection Agency (EPA)/National Institutes of Health (NIH) mass spectral library (ver. 2.0). A GC chromatogram was used to quantify the volatile compounds, and mass spectrometry was used to identify the volatile compounds. Results of the analyses are expressed as percentages of the total chromatographic area.

### Statistical analysis

All data are expressed as mean±standard error. The data except the sensory traits were analyzed by analysis of variance using the general linear model procedure in the SAS software (SAS Institute, Cary, NC, USA). A linear mixed model was used to analyze sensory traits with the diet treatment as a fixed effect and session and panelist as random effects. The threshold for significance was p≤0.05, and tendencies are indicated by 0.05<p≤0.10.

## RESULTS AND DISCUSSION

### Growth performance and digestibility

The concentrate intake was higher (p<0.01) in the glycerol-inclusion group than in the control group (Table 2). The average daily gain and feed efficiency (gain to feed ratio) were not affected (p>0.05) by glycerol inclusion. An increased DM intake with feeding total mixed ration (TMR) containing 5% crude glycerin inclusion was observed during prepartum in dairy cattle, but not during the postpartum period with feeding TMR containing 3.3% crude glycerin [28]. Increasing the crude glycerin to 4%, 8%, or 12% in concentrates in the form of pellets also did not affect the feed intake or growth performance in Holstein bulls [2]. Feeding dry glycerin at 250 g/d as a top dressing to early postpartum Holstein dairy cows did not affect feed intake [7]. The reason for the increased feed intake observed in the present study may have been the use of purified glycerol rather than crude glycerin, since most of crude glycerin inclusion did not affect intake as cited above. Crude glycerin may contain small quantities of salts and methanol, which can affect the palatability of the final glycerol product, as suggested by Chung et al [7]. Fisher et al [29] suggested that glycerol can act as an appetite stimulant in Holstein cows. In the glycerin inclusion concentrate, glycerol was present in place of molasses. Thus, glycerol may have a stronger appetite-stimulating effect than molasses in the present study. Glycerol inclusion did not affect (p>0.05) the apparent digestibility of DM, organic matter, or fat (Table 2).

### Rumen fermentation and blood parameters

The pH and the concentrations of acetate, propionate, butyrate, total VFA and NH_3_-N in rumen fluid were not affected (p>0.05) by glycerol inclusion (Table 3). The ratio of acetate to propionate tended to be lower (p = 0.06) in the glycerol group than in the control group at the 16th week, but not at other periods. In the rumen, glycerol can be fermented to propionate, which can act as glucogenic precursor in ruminants [8]. In the current study, the actual average glycerol intake in the glycerol inclusion group was 253 g/d, and this relatively small amount of glycerol inclusion may be insufficient to significantly affect the ruminal propionate production. In a previous study, glycerin supplementation at 430 g/d increased the ruminal propionate and total VFA concentration in Holstein cows [30]. In addition, the sugars in molasses is also converted in part to propionate as glycerol is converted to propionate [31]. Thus, replacement of portion of molasses with glycerol in glycerol group may be another explanation for no difference in propionate concentrations between treatments.

The serum glucose concentrations tended to be higher (p≤0.10) in the glycerol inclusion group than in the control group at the 16th and 20th weeks, but not at other weeks (Table 4). In the rumen, glycerol can be converted to propionic acid, which is in turn absorbed into the rumen wall [9]. Propionic acid and glycerol can then be converted to glucose through gluconeogenesis [9]. Thus, the increased tendency of serum glucose concentrations in the glycerol group may have been due to increased glucose production via gluconeogenesis. The increased tendency of blood glucose in glycerol group may reflect attenuation effect of substituting glycerol with molasses. Glycerin supplementation from parturition to 21 d postpartum of Holstein dairy cows tended to increase the blood glucose concentrations during the second week of lactation [7].

We observed increased concentrate intake with glycerol inclusion. We measured circulating ghrelin concentrations since ghrelin is known to increase food intake in rats [32]. The plasma ghrelin concentrations were not affected (p>0.05) by glycerol inclusion (Table 4). However, exogenous ghrelin infusion did not affect the feed intake in sheep [33]. Thus, *ad libitum* feeding with proper nutritional levels in the diet may not significantly affect the circulating ghrelin concentrations in beef cattle.

### Carcass characteristics and physio-chemical composition and sensory traits in the *longissimus thoracis*

Carcass weight was not affected (p>0.05) by glycerol inclusion (Table 5). All carcass traits, including the LM area, backfat thickness, marbling score, meat color, quality grade, and yield grade, were not affected (p>0.05) by glycerol inclusion (Table 5). A previous study showed that dietary crude glycerin replacing other ingredients up to 12% did not affect the carcass weight, backfat thickness, or LM area [2]. Another study showed that supplementation of crude glycerin up to 28% DM did not affect the meat color, fat color, or shear force of LM in Nellore young bulls on pasture [34]. Therefore, the level of glycerol inclusion was assumed to be insufficient to affect the carcass characteristics in our study.

The chemical composition of the LT, including crude fat, crude protein, and moisture, were not affected (p>0.05) by glycerol inclusion (Table 6). Similarly, previous research has shown that crude glycerin supplementation did not affect the moisture, fat, or protein contents in the LM of cattle [35]. The physicochemical characteristics of the LT, including CIE L* (brightness), a* (redness), b* (yellowness), and shear force, were not affected (p>0.05) by glycerol inclusion. This is consistent with previous studies showing that crude glycerin supplementation did not affect the CIE values of beef in Nellore bulls [34]. In the present study, the pH in the LT was lower (p = 0.001) in the glycerol group (pH = 5.56) than in the control group (pH = 5.64; Table 6). Similarly, a previous study found that the pH decreased with the addition of dietary glycerin in the LM of Nellore bulls [34]. San Vito et al [34] suggested that this decrease may have been due to increased energy reserves (e.g. glycogen) that maintain anaerobic metabolism, resulting in the production of lactic acid, which can lower the meat pH. However, the present study showed no difference in the glycogen content between the groups. Glycolytic potential is calculated based on the contents of glycogen, glucose-6-phoshpate, glucose, and lactic acid, and the muscle glycolytic potential affects the slaughter pH and ultimate pH in cattle [36]. Another explanation for the decreased meat pH associated with glycerin inclusion may be a change in the glycolytic potential. Although the explanation for the lower pH in the LT of the glycerol group is unclear, the LT pH values of both groups in the present study were within the normal pH range (5.4 to 5.7) of unstressed animals.

The reducing sugar and glycogen contents of the LT were not affected (p>0.05) by glycerol inclusion (Table 6). Glycerol can be converted into glucose via gluconeogenesis, mainly in the liver [9]. Higher glucose production may contribute to glycogen deposition in the liver and muscle [11]. In the present study, the actual glycerol supply was 253 g/d in the glycerol group, which is much less than the 860 g/d of glycerol that was provided through top-dressing in a study by DeFrain et al [30], in which no difference in plasma glucose concentration was observed. Conversely, another study showed that feeding glycerol at 1 L, 2 L, or 3 L by drenching through an esophageal pump in cows increased the blood glucose concentrations by 16%, 20%, and 25%, respectively, 30 minutes after dosing [37]. Thus, the inclusion of relatively low levels of glycerol in the concentrate may not significantly affect the reducing sugar and glycogen contents. We have found no previous study evaluating the effects of glycerol intake on reducing sugars in the LM of beef cattle. Previously, we found that the fat content in the LT is highly related to the reducing sugar contents in pooled data from Korean cattle, Holstein cattle, and Angus steers [26]. Therefore, no difference in the crude fat content of the LT might be due to the similar contents in the reducing sugar and glycogen of the LT between the two groups.

All sensory traits in the LT, including appearance, odor, taste, flavor, tenderness, juiciness, and overall acceptance, were unaffected (p>0.05) by glycerol inclusion (Table 6). Similarly, no significant differences in tenderness, juiciness, or flavor were observed in the LM of Purunã bulls fed on diets containing up to 18% glycerin [38]. Sensory traits, including tenderness, are positively related to the intramuscular fat content in cattle [39]. Thus, the lack of an effect of glycerol inclusion on the intramuscular fat content may partly explain the absence of differences in sensory traits between the two groups in the present study.

### Volatile compound profiles in the *longissimus thoracis*

Aldehyde profiles (pentanal, hexanal 2-methyl-propanal, etc.) were not affected (p>0.05) by glycerol inclusion (Table 7). During cooking, the main reactions that produce aromatic volatile compounds are the Maillard reaction between reducing sugars and amino acids, as well as the thermal degradation of lipids [12]. Aldehydes are mainly formed by the oxidation of unsaturated fatty acids, such as oleic acid, linoleic acid, and linolenic acid, during cooking [12]. The absence of changes in the reducing sugar content or fat content may explain why volatile compounds did not differ between the two groups. The hydrocarbon profiles also did not differ (p>0.05) between the two groups. Hydrocarbon is known to be produced from lipid oxidation catalyzed by heme compounds such as hemoglobin and myoglobin [12]. The profiles of ketones, including 2-propanaone, 2-butanone, and 3-hydroxy-2-butanone, did not differ (p>0.05) between the two dietary groups. Interestingly, the 2,3-butanedione (buttery) and 2,3-octanedione (oxidized fat flavor) contents were higher (p<0.05) and lower (p<0.05) in the glycerol group than in the control group, respectively. However, these differences may not significantly influence the sensory traits since the levels of these compounds were very low (less than 1.0%).

## CONCLUSION

The inclusion of purified glycerol as a replacement for DDGS in the finishing diet did not affect growth performance, rumen fermentation parameters, and carcass quality in Korean cattle, which was not consistent with our hypothesis. The DDGS often reveals variations in nutrient composition among sources of DDGS, whereas the purified glycerol has a consistent energy level. Thus, purified glycerol with uniform energy levels could be used in beef cattle as a substitute for other energy sources such as DDGS, which may have variation in compositions, depending on the price.

## Figures and Tables

**Table 1:** Ingredients of concentrates and chemical composition of the experimental diets

Item	Concentrate	

	Control	Glycerol
Ingredients (% of DM)		
Ground wheat	20.36	22.54
Steamed flaked corn	18.29	18.41
Ground corn	9.40	8.65
Distiller’s dried grains with solubles	9.97	6.02
Wheat bran	6.41	6.11
Palm kernel meal	6.76	6.81
Copra meal	4.88	4.91
Molasses	3.62	0.91
Whole cottonseed	3.97	4.00
Corn gluten feed	4.65	5.07
Rice bran	2.00	4.04
Soyhull	2.05	2.87
Palm oil	1.48	0.87
Alfalfa pellet	0.97	0.98
Cottonseed hull	0.96	0.96
Purified glycerol	0.40	3.57
Condensed molasses solubles	0.22	0.00
Sodium bentonite	0.29	0.30
Magnesium oxide	0.36	0.36
Sodium bicarbonate	0.91	0.89
Salt	0.25	0.25
Urea	0.00	0.21
Limestone	1.57	1.06
Mineral-vitamin premix1)	0.22	0.23
Chemical composition of concentrate (% of DM)		
DM	89.6	89.7
CP	14.5	14.5
EE	4.99	4.70
Ash	6.77	6.53
NDF	31.5	31.4
ADF	14.2	13.6
Calcium	0.84	0.72
TDN2)	85.4	85.2
ME3) (Mcal/kg DM)	3.30	3.29
Chemical composition of rice straw (% of DM)		
DM	93.8	-
CP	5.1	-
EE	0.76	-
Ash	13.1	-
NDF	75.1	-
ADF	52.2	-

DM, dry matter; CP, crude protein; EE, ether extract; NDF, neutral detergent fiber; ADF, acid detergent fiber; TDN, total digestible nutrient; ME, metabolizable energy; DE, digestible energy.

1) Provided following nutrients per kg of additive (Grobic-DC, Bayer Health Care, Leverkusen, Germany): Vit. A, 2,650,000 IU; Vit. D_3_, 530,000 IU; Vit. E, 1,050 IU; niacin, 10,000 mg; Mn, 4,400 mg; Zn, 4,400 mg; Fe, 13,200 mg; Cu, 2,200 mg; I, 440 mg; Co, 440 mg.

2) TDN (%) = non-fiber carbohydrate+CP+(fatty acids×2.25)+NDF–7 [18].

3) ME = (1.01×DE^4)^–0.45)+0.0046×(EE–3) [18].

4) DE = 0.04409×TDN [18].

**Table 2:** Intake, performance, and nutrient digestibility of Korean cattle steers fed the control or glycerol inclusion diet

Item	Control	Glycerol	SEM	p-value
Initial BW (kg)	647	648	10.49	0.97
Final BW (kg)	731	737	12.19	0.58
Average daily gain (kg/d)	0.57	0.61	0.03	0.55
Concentrate intake (kg of DM/d)	6.2	7.1	0.18	0.001
Rice straw intake (kg of DM/d)	0.9	0.9	0.00	-
Total feed intake (kg of DM/d)	7.1	8.0	0.16	0.001
Feed efficiency (kg gain/kg DM intake)	0.08	0.076	0.003	0.59
Digestibility (%)				
DM	56.6	58.1	1.14	0.47
Organic matter	61.2	62.7	0.98	0.42
Fat	84.0	81.8	1.46	0.43

n = 10 per group.

SEM, standard error of the mean; BW, body weight; DM, dry matter.

**Table 3:** Ruminal pH and volatile fatty acid and NH_3_-N concentrations of Korean cattle steers fed the control or glycerol inclusion diet

Item	Control	Glycerol	SEM	p-value
pH				
4th wk	6.26	6.20	0.07	0.64
8th wk	6.41	6.43	0.07	0.88
12th wk	6.41	6.52	0.08	0.50
16th wk	6.74	6.73	0.09	0.94
20th wk	6.67	6.83	0.08	0.37
Acetate (mM)				
4th wk	60.6	56.6	1.84	0.30
8th wk	57.0	55.8	2.32	0.84
12th wk	67.8	61.3	3.12	0.19
16th wk	57.4	49.9	2.96	0.30
20th wk	61.9	51.4	3.47	0.17
Propionate (mM)				
4th wk	18.7	25.3	2.69	0.26
8th wk	20.7	21.6	1.79	0.84
12th wk	22.7	23.1	1.84	0.94
16th wk	16.6	21.7	1.86	0.16
20th wk	18.5	14.9	1.21	0.20
Butyrate (mM)				
4th wk	17.7	16.9	1.21	0.72
8th wk	15.5	15.0	0.99	0.83
12th wk	18.6	16.8	1.18	0.22
16th wk	16.0	17.2	1.56	0.74
20th wk	16.3	15.1	1.21	0.61
Total VFA (mM)				
4th wk	101.8	103.9	4.16	0.84
8th wk	98.1	97.6	4.51	0.96
12th wk	114.9	106.8	5.24	0.44
16th wk	94.7	93.5	5.52	0.92
20th wk	101.2	85.9	5.93	0.24
Acetate:propionate ratio				
4th wk	3.29	2.77	0.19	0.16
8th wk	3.07	2.71	0.18	0.37
12th wk	3.14	3.11	0.23	0.96
16th wk	3.58	2.74	0.21	0.06
20th wk	3.39	3.56	0.09	0.39
NH_3_-N (mg/100 mL)				
4th wk	11.5	8.22	1.72	0.31
8th wk	7.33	6.63	1.23	0.80
12th wk	10.5	8.37	1.48	0.46
16th wk	7.95	6.63	1.08	0.62
20th wk	9.39	7.95	1.22	0.53

n = 10 per group.

SEM, standard error of the mean; VFA, volatile fatty acid.

**Table 4:** Serum glucose and plasma ghrelin concentrations at 3 h post-feeding of Korean cattle steers fed control or glycerol inclusion diet

Item	Control	Glycerol	SEM	p-value
Glucose (mg/dL)				
4th wk	59	55.7	1.48	0.36
8th wk	61.2	59.9	0.99	0.51
12th wk	62.2	62.7	0.80	0.76
16th wk	69.9	72.2	0.67	0.10
20th wk	71.7	75.3	0.94	0.055
Ghrelin (ng/mL)				
16th wk	298	290	4.03	0.39
20th wk	295	300	5.73	0.58

n = 10 per group.

SEM, standard error of the mean.

**Table 5:** Carcass weight and carcass characteristics of Korean cattle steers fed the control or glycerol inclusion diet

Item	Control	Glycerol	SEM	p-value
Carcass weight (kg)	433	436	7.73	0.87
*Longissimus* muscle area (cm^2^)	96.4	97	2.29	0.92
Backfat thickness (mm)	12.4	11.8	0.50	0.47
Marbling score1)	5.70	5.50	0.34	0.82
Yield index2)	65.8	66.2	0.36	0.59
Meat color3)	4.60	4.80	0.11	0.34
Fat color4)	3.00	3.00	0	-
Texture5)	1.10	1.10	0.07	1.00
Maturity6)	2.30	2.50	0.11	0.34
Yield grade7)	2.10	1.90	0.10	0.34
Quality grade8)	2.40	2.50	0.17	0.82

n = 10 per group.

SEM, standard error of the mean.

1) Marbling score: 1, devoid; 9, very abundant.

2) Yield index = 68.184–[0.625×backfat thickness (mm)]+[0.130×*Longissimus* muscle area (cm^2^)]–[0.024×carcass weight (kg)] [20].

3) Meat color: 1, bright red; 7, dark red.

4) Fat color: 1, white; 7, yellowish.

5) Texture: 1, very fine; 3, very coarse.

6) Maturity: 1, youthful; 9, mature.

7) Yield grade: A = 1, B = 2, C = 3.

8) Quality grade: 1^++^ = 1, 1^+^ = 2, 1 = 3, 2 = 4, 3 = 5.

**Table 6:** Chemical and physico-chemical compositions, reducing sugar and glycogen contents, and sensory traits of the longissimus thoracis from Korean cattle steers fed the control or glycerol inclusion diet

Item	Control	Glycerol	SEM	p-value
Chemical composition (%)				
Moisture	61.9	60.0	0.99	0.46
Crude protein	19.0	19.1	0.27	0.90
Crude fat	16.1	17.9	1.10	0.51
Physico-chemical composition				
CIE L^*^	43.5	45.9	0.76	0.18
CIE a^*^	30.7	30.3	0.42	0.67
CIE b^*^	25.9	26.5	0.2	0.13
pH	5.64	5.56	0.01	0.001
Shear force (kg)	7.05	7.11	0.37	0.94
Reducing sugar content (%)	0.34	0.34	0.01	0.89
Glycogen content (mg/g)	5.12	4.08	0.48	0.37
Sensory traits1)				
Appearance	5.88	5.88	0.04	1.00
Odor	5.97	6.09	0.15	0.76
Taste	6.06	6.27	0.24	0.71
Flavor	6.12	6.18	0.21	0.92
Tenderness	5.18	6.03	0.32	0.19
Juiciness	5.76	5.85	0.27	0.89
Overall acceptance	6.09	6.06	0.20	0.95

n = 10 per group.

SEM, standard error of the mean; CIE, Commission Internationale de l’Eclairage.

1) The score was evaluated by ten semi-trained panelists (1, dislike extremely; 5, neither dislike nor like; 9, like extremely).

**Table 7:** Volatile compound levels (%) of the *longissimus thoracis* from Korean cattle steers fed the control or glycerol inclusion diet

Volatile compound	Control	Glycerol	SEM	p-value
Aldehyde				
Propanal	1.64	1.33	0.27	0.41
Pentanal	5.77	5.93	1.17	0.90
Butanal	0.26	1.08	0.38	0.32
Hexanal	13.7	12.8	2.35	0.70
Heptanal	1.04	1.32	0.28	0.48
Octanal	0.42	0.56	0.11	0.40
Nonanal	0.43	0.40	0.06	0.70
2-Methyl propanal	4.19	4.58	0.33	0.33
3-Methyl butanal	1.09	1.00	0.07	0.15
Benzaldehyde	0.78	0.92	0.07	0.18
Hydrocarbon				
Methanethiol	8.07	9.62	1.10	0.33
2-Butene	0.47	0.58	0.05	0.33
Carbon disulfide	0.84	1.09	0.10	0.18
Methyl acetate	0.04	0.01	0.02	0.37
Butane	1.53	1.63	0.11	0.72
n-Pentane	2.30	1.75	0.27	0.24
Hexane	0.60	0.49	0.07	0.30
Heptane	0.22	0.18	0.02	0.39
Octane	0.37	0.25	0.04	0.10
Ketone				
2-Propanone	44.8	38.9	3.18	0.12
2-Pentanone	0.18	0.13	0.02	0.07
2-Heptanone	0.22	0.20	0.02	0.47
2-Butanone	2.05	1.87	0.13	0.34
2,3-Butanedione	0.38	0.61	0.06	0.04
3-Hydroxy-2-butanone	3.89	7.08	1.16	0.19
2,3-Octanedione	0.67	0.45	0.09	0.04
Alcohol				
Isopropyl alcohol	0.66	0.52	0.07	0.30
Other				
Dimethyl sulfide	0.96	0.98	0.08	0.93
1,3,5-Cycloheptatriene	0.47	0.56	0.04	0.13
Ethyl-acetate	1.86	3.13	0.45	0.18

n = 10 per group.

SEM, standard error of the mean.
